# Downregulation of *DLC-1* Gene by Promoter Methylation during Primary Colorectal Cancer Progression

**DOI:** 10.1155/2013/181384

**Published:** 2012-12-23

**Authors:** Haixia Peng, Feng Long, Zhiyuan Wu, Yimin Chu, Ji Li, Rong Kuai, Jing Zhang, Zhihua Kang, Xinju Zhang, Ming Guan

**Affiliations:** ^1^Department of Gastroenterology, Shanghai Changning District Cental Hospital, Shanghai 200336, China; ^2^Department of Respiratory Medicine, Huashan Hospital, Shanghai Medical College, Fudan University, Shanghai 200040, China; ^3^Department of Laboratory Medicine, Huashan Hospital, Shanghai Medical College, Fudan University, Shanghai 200040, China; ^4^Central Laboratory, Huashan Hospital, Shanghai Medical College, Fudan University, Shanghai 200040, China; ^5^Department of Nursing, Huashan Hospital, Shanghai Medical College, Fudan University, Shanghai 200040, China

## Abstract

*Purpose*. *DLC-1 *is a tumor suppressor gene frequently silenced in human cancers. However, the pathogenicity of *DLC-1 *epigenetic silencing in the mucosa-adenoma-carcinoma transformation process of colorectal cancer (CRC) has not been studied. *Methods*. Promoter methylation status of *DLC-1* was evaluated in 4 human CRC cell lines, 48 normal mucosa, 57 adenomas, and 80 CRC tissues with methylation-sensitive high-resolution melting analysis (MS-HRMA), while the mRNA expression was examined by qPCR. HRMA was utilized to detect the *KRAS* codon 12, 13 and *BRAF* V600Emutations. *Results*. Partial (1%–10%) and extensive (10%–100%) *DLC-1* promoter methylations were observed in 10% and 0% of normal mucosa, 46% and 14% of adenomas, and 60% and 36% of CRCs, respectively. The promoter methylation of *DLC-1 *was related with the reduction of gene expression and the advanced Duke's stages (Stage C and D). *DLC-1* promoter methylation and *KRAS* mutations are common concurrent pathological alternations. *Conclusions*. Epigenetic alternation plays a key role in the transcriptional silencing of *DLC-1*. It is also an independent risk factor related to the carcinogenesis of colorectal tumors and spans over its pathogenesis process. Therefore, *DLC-1* promoter methylation quantitation may have a promising significance in the evaluation and management of CRC patients.

## 1. Introduction

Colorectal cancer (CRC) arises as a consequence of genetic and epigenetic alterations. Somatic mutations and epigenetic alterations have been frequently observed in CRC and are considered to be the driver factors of colorectal tumorigenesis [1]. The neoplastic progression of CRCs mostly follows a mucosa-adenoma-carcinoma step-by-step pattern, which is accompanied by an accumulation of successive genetic alterations (e.g., *APC*, *KRAS/BRAF*, and *TP53*) [2–5]. Widespread promoter CpG island methylation, also referred to as the CpG island methylator phenotype (CIMP), has been extensively studied in CRC. CIMP-high CRC significantly correlates to microsatellite instability, *BRAF* mutation and low *TP53* mutation rate, while CIMP-low tumors present a higher rate of *KRAS* mutation [6]. Despite these extensive researches, the molecular mechanisms underlying the colorectal mucosa-adenoma-carcinoma transformation process and the progression of CRC are still not fully understood.

Deleted in liver cancer-1, *DLC-1* gene is a recently identified tumor suppressor gene. It is located on chromosome 8p21-8p22 and affected in multiple cancers. DLC-1 is a regulator of the Rho family of small GTPases [7–12]. The principal function of DCL-1 is to catalyze the conversion of active guanosine triphosphate- (GTP-) bound RhoA to the inactive guanosine biphosphate- (GDP-) bound form. DLC-1 is recurrently downregulated or inactivated by epigenetic mechanisms in the initiation and progression of cancers [13].

Ullmannova and Popescu [14] first reported the downregulation of DLC-1 mRNA expression in CRCs utilizing cancer-profiling arrays. Moreover, aberrant methylation of CpG islands in the promoter region of *DLC-1* is a common mechanism leading to the transcriptional silencing [15–18], suggesting *DLC-1* methylation is associated with the downregulation of this gene in CRC and *DLC-1* may be a potential tumor suppressor gene. However, methylation and mRNA expression status of *DLC-1* and its role in the adenoma-carcinoma progression have not been defined.

To study the role of *DLC-1* promoter methylation in the colorectal carcinogenesis routing down the adenoma-carcinoma process, here we quantified the methylation status and mRNA expression of *DLC-1* and assessed its relation to various clinicopathological parameters and molecular features, especially the mutation status of *KRAS* and *BRAF* in 185 colorectal tissue samples from different disease stages.

## 2. Materials and Methods

### 2.1. Patients

Eighty colorectal carcinomas, 57 adenomas, and 48 samples of adjacent histologically normal mucosa were collected from the Department of Gastroenterology at Shanghai Changning District Cental Hospital and Huashan Hospital. Tissue samples were frozen within 2 hours of removal and then stored at −80°C. All the tumors contained more than 80% tumor cells as confirmed by histological examination of sequential sections. The patient's gender, age, Duke's stage, tumor differentiation, and tumor size and location were obtained from surgical and pathological records. In compliance with the Helsinki Declaration of 1975 as revised in 1996, this study was approved by the Institutional Review Board of Shanghai Changning District Center Hospital.

### 2.2. Cell Lines

Four human colorectal carcinoma cell lines (SW480, LoVo, LS 174T, and COLO 320) were obtained from the American Type Culture Collection (ATCC, Manassas, VA, USA) cell bank. The SW480 and LS 174T cells were cultured in RPMI 1640 medium; the LoVo and COLO 320 cells were cultured in F12K medium (Gibco, Grand Island, NY, USA). Both the mediums were supplemented with 10% fetal bovine serum (Sigma, St. Louis, MO, USA) and incubated in 5% CO_2_ at 37°C.

### 2.3. Methylation-Sensitive High-Resolution Melting Analysis (MS-HRMA) of *DLC-1*

Genomic DNA was extracted from each tumor tissue sample and cell line using QiaAmp NDA extraction kit (Qiagen, Valencia, CA, USA) according to the manufacturer's instructions. Genomic DNA was then modified by sodium bisulfite using EZ DNA Methylation kit (Zymo Research, Irvine, CA, USA) according to the manufacturer's instructions. A methylated reference sample, the CpGenome Universal methylated human male genomic DNA (Chemicon, Billerica, MA, USA), and genomic DNA isolated from the peripheral blood mononuclear cells of a healthy male individual were subjected to the bisulfite modification procedure and used as control standards. The methylated reference was then diluted with the unmethylated control in 0%, 1%, 10%, 30%, 50%, 80%, and 100% ratios to carry out the standard curve for the MS-HRMA. The PCR amplification and HRMA were then performed using the methylation specified primers (MS-HRM-F, MS-HRM-R, Table 1). The 20 *μ*L volume master mix contained 10 *μ*L Premix Taq Hot Start Version (2X) (TaKaRa BIO, Shiga, Japan), 1 *μ*L forward primer (5 *μ*M), 1 *μ*L reverse primer (5 *μ*M), 1 *μ*L SYTO 9 dye (30 *μ*M) (Invitrogen, Carlsbad, CA, USA), 6 *μ*L deionized distilled water, and 1 *μ*L DNA template (15–25 ng/*μ*L). The mix was subjected to PCR on a Rotor-Gene Q real-time platform (Qiagen, Valencia, CA, USA). The samples were denaturated at 95°C for 2 min, followed by 40 cycles of 95°C for 30 sec, 58°C for 30 sec, and 72°C for 30 sec. After PCR, the products were again denaturated at 95°C for 2 min and cooled down to 40°C for 2 min to form the heteroduplex. HRMA was then performed at 0.2°C/s from 50°C to 95°C. Each sample was tested in duplicate. The HRMA and data interpretation were performed using Rotor-Gene Q 1.7 software. A differential profile was then evaluated for each sample by comparing fluorescence at the melting point with the fluorescence of the unmethylated control. There was a linear correlation between the differential fluorescence and the dilution of methylated DNA.

### 2.4. Bisulfite DNA Sequencing

PCR primers were designed as previously described by Guan et al. [19] to amplify a 292 bp region of the *DLC-1* promoter that encompasses 35 CpGs (Figure 2(a)). The PCR product was then subcloned into the pMD19-T expression vector by using a TA Cloning Kit (TaKaRa BIO, Shiga, Japan). We selected four clones from each plate and sent the recombinant plasmids to MAP Biotech (Shanghai, China) to complete the bisulfite DNA sequencing procedure.

### 2.5. Quantitative Reverse Transcription PCR

Total RNA was extracted using Trizol (Invitriogen, Carlsbad, CA, USA) according to the manufacturer's instructions. Reverse transcription reactions were carried out on 1 ug total RNA with the PrimeScript RT reagent kit (TaKaRa BIO, Shiga, Japan) using the random hexamer primers. The real-time PCR was then performed using SYBR Premix DimerEraser kit (TaKaRa, Shiga, Japan). The 2^−ΔΔCt^ method was used to calculate the relative fold difference of *DLC-1* mRNA expression in all the cancerous tissue samples compared to the average ratio of normal samples. A twofold decreased expression was considered significant. All the reactions were carried out on the ABI PRISM 7500 real-time PCR system (Applied Biosystems, Foster City, CA, USA). The primers used (DLC-1F and DLC-1R; GAPDH-F and GAPDH-R) were listed in Table 1. The experiments were of strict compliance with the MIQE (Minimum Information about Quantitative Real-Time PCR Experiments) guidelines.

### 2.6. HRMA Analysis of **KRAS ** Codon 12, 13 and *BRAF* V600E Mutations

Genomic DNA was extracted from each tumor tissue sample and cell line using QiaAmp DNA extraction kit (Qiagen, Valencia, CA, USA) according to the manufacturer's instructions. AsPC-1 (*KRAS* condon 12, 13 homozygous mutation), HT29 (*BRAF* V600E heterozygous mutation), and SW480 cells (*BRAF* V600E wild-type and *KRAS* wild-type, homozygous) were used as positive controls for *KRAS* codon 12, 13 mutation, *BRAF* V600E mutation, and negative controls, respectively. The DNA was then amplified by the specific primers (KRAS-F and KRAS-R; BRAF-F and BRAF-R, Table 1) [20, 21], producing small amplicons for the HRMA. The PCR mix preparation and the HRMA procedure were carried out with the identical methodology as described in the MS-HRMA of DLC-1 section. For each sample, the normalized melting curves were evaluated and then compared with the mutant and wild-type controls in a deduced difference plot. The samples with distinct melting curves compared with the wild-type allele were recorded as positive.

### 2.7. Statistical Analysis

The association of *DLC-1* methylation and mRNA expression with patients' clinical and genetic variables was analyzed with the *χ* ^2^ test. *P* < 0.05 was considered statistically significant.

## 3. Results

### 3.1. MS-HRMA Analysis of **DLC-1 ** Methylation

**Figure 1:**
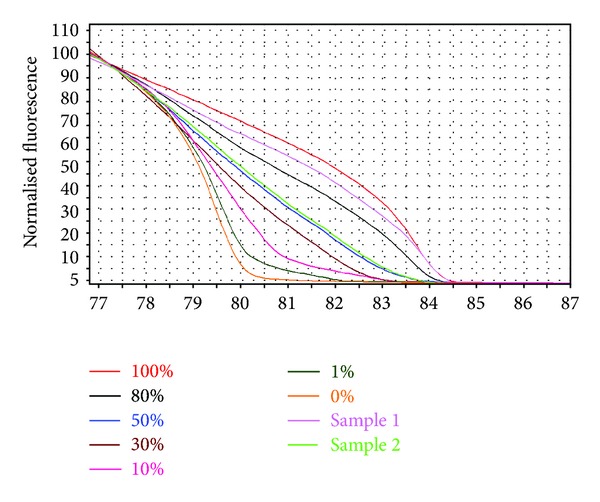
MS-HRMA was used for quantification of *DLC-1* methylation status. Profile of fluorescence obtained at the melting temperature for serial dilutions of methylated DNA (from 100 to 0%) and two samples.

MS-HRMA was designed to examine the methylation status in promoter regions of *DLC-1* in four colon cancer cell lines and various tumor and adjacent normal tissues (Figure 1). When methylation level higher than >1% was considered methylation positive, *DLC-1* methylation was observed in 5/48 (10%) of normal mucosa, 26/57 (46%) of adenomas, and 48/80 (60%) of CRCs. However, extensive methylation (methylation level of 10–100%) was observed in only 8/57 (14%) of adenomas, 29/80 (36%) of CRCs, and 0% of the normal mucosa (Table 2). Three of four colon (75%) carcinoma cell lines were methylated in the *DLC-1* promoter region at various levels (SW480: 76.9%, LoVo: 62.5%, and LS174T: 44%), whereas no CpG site was methylated in COLO 320 cells.

### 3.2. Bisulfite DNA Sequencing

**Figure 2:**
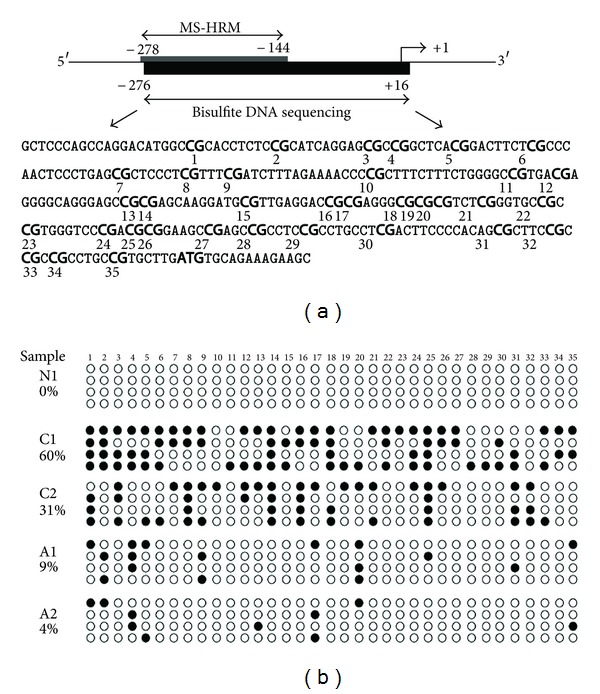
Bisulfite sequencing of the CpG island in *DLC-1* promoter region. (a) Schematic depiction of the *DLC-1* promoter-associated CpG island, which spans the region from −278 to +16 (the transcription start site ATG as +1). Regions analyzed by MS-HRM and bisulfite genomic sequencing (Bis-*DLC-1*) are shown. The Bis-*DLC-1* region encompassed 292 bp and contained 35 CpG dinucleotides. (b) Methylation patterns of the Bis-*DLC-1* region of the  *DLC-1* CpG island in a normal mucosa tissue (N1), two adenomas (A1 and A2), and two CRCs (C1 and C2) samples that were identified by MS-HRM as methylated. Methylated and unmethylated CpG sites are shown as solid circles and open circles, respectively.

Bisulfite DNA sequencing was employed to determine the comprehensive methylation pattern of the 5′-CpG islands in the *DLC-1* promoter. Five CRCs and five adenoma samples that were identified by MS-HRMA as extensive methylation and partial methylation, respectively, and two normal mucosa tissue samples were amplified with a 292 bp fragment in the *DLC-1* promoter, covering 35 CpG sites. Bisulfite DNA sequencing confirmed the CpG islands in the normal sample unmethylated; all five CRC samples were frequently methylated. The tumor samples shared some common methylation sites, while the overall methylation patterns were distinct. Consistent with the MS-HRMA data, the CpG island exhibited that more CpG sites were methylated in clones obtained from CRCs than those from adenomas. We found the methylation of *DLC-1* promoter more frequently methylated in the CRC samples (C1, 60% and C2, 31%) than in the adenomas (A1, 9% and A2, 4%) (Figure 2(b)).

### 3.3. Correlation of *DLC-1* Methylation with Clinical and Pathological Features

The correlations between the methylation status of *DLC-1* and clinical features in the CRC and adenoma patients were presented in Table 3. *DLC-1* methylation was significantly higher in the CRC tissues with advanced Duke's stage (Stage C, versus Stage A + B, *P* = 0.025; Stage D, versus Stage A + B, *P* = 0.002). However, no significant difference in the promoter methylation level was observed between Stages C and D (*P* = 0.902). Other than this, there were no significant associations between *DLC-1* methylation status and other clinical factors. Among adenomas, there were no significant associations between *DLC-1* methylation status and clinical parameters.

### 3.4. Correlation of *DLC-1* Methylation with *DLC-1* mRNA Expression

We determined the mRNA expression of *DLC-1* gene in 4 colon cancer cell lines, normal mucosa, adenomas, and CRC tissues. *GAPDH* mRNA was used as a housekeeper for cDNA integrity. *DLC-1* mRNA expression was detected in Colo320 cells but not in SW480, LoVo, and LS 174T cell lines which harbor methylation in the promoter region of *DLC-1*. *DLC-1* mRNA expression was observed in 41/48 (85%) of normal mucosa specimens. Of the 57 adenomas and 80 CRC tissues, *DLC-1* mRNA was downregulated in 30/57 (52%) and 63/80 (79%) samples, respectively (Table 2). Since very low level of methylation could not lead to significant downregulation of gene expression, we considered 10% methylation of *DLC-1* promoter region as the cutoff. There was a correlation between *DLC-1* mRNA expression reduction and extensive promoter methylation status (Table 4, *P* = 0.022). Our results suggest that the reduction or loss of *DLC-1* mRNA expression was related to the extensive methylation in *DLC-1* promoter.

### 3.5. Correlation of *DLC-1* Methylation with KRAS and BRAF Mutations

We found *KRAS* mutations in 4/48 (8%) normal mucosa, 10/57 (18%) adenomas, and 32/80 (40%) CRCs (Table 2). When we examined the association of KRAS mutations to *DLC-1* methylation, a statistically significant correlation was observed only in the CRCs (*P* = 0.010), but not in adenomas (*P* = 0.889). Hereafter, we investigated the association between extensive methylation of *DLC-1* promoter and mutations in *KRAS* codon12, 13. In terms of CRCs, 62% (18/29) of tumors with extensive *DLC-1* methylation showed *KRAS* mutations, while *KRAS* mutation alterations were present in only 32% (6/19) of tumors with partial methylation and 25% (8/32) of tumors with unmethylated *DLC-1* promoter. These data demonstrated that *KRAS* mutations significantly correlated with extensive *DLC-1* promoter methylation in CRCs. However, we did no further investigation in the correlation between BRAF mutation and *DLC-1* methylation because only 4 of 185 tissues turned out to be *BRAF* V600E positive from the HRMA, limiting the statistical power of the data.

## 4. Discussion

The identification of genes contributing to the development of colon cancer is critical to the understanding of molecular mechanisms of carcinogenesis and may provide new strategies for clinical therapy. A new candidate tumor suppressor gene, *DLC-1*, was first identified as a rat p122RhoGAP homolog [10]. This gene is diminished or silenced in various types of human cancers as well as in metastatic cells compared to nonmetastatic cells [7–9, 11, 12]. *DLC-1* has not been extensively studied in CRC. Moreover, the transcriptional regulation of *DLC-1* gene expression through epigenetic mechanisms has not been investigated in the normal adenoma-carcinoma sequence. The relationships between *DLC-1* methylation status and clinic pathological variables in CRC remain to be elucidated.

In this study, we used MS-HRMA to detect *DLC-1* promoter methylation in 48 normal mucosa, 57 adenomas, 80 CRC tissue samples, and 4 CRC cell lines. The methylation status of *DLC-1* did not show a strong correlation with the widely accepted risk factors of CRC including age and sex (estrogen) [22]. We found the mRNA expression of DLC-1 was decreased when *DLC-1* promoter was methylated in the cancerous tissues. Moreover, partial methylation was frequently observed in adenomas as well as CRC. Extensive methylation was primarily observed in CRCs but less prevalent in adenomas. In addition, by quantifying the methylation status in *DLC-1* promoter, we also found an accumulation of aberrant methylation following the adenoma-carcinoma sequence. During this stepwise progression, the methylation level of the *DLC-1* promoter region increased gradually and the existence of cytosine methylation expanded widely. None of the normal adjacent mucosa specimens showed extensive methylation in the *DLC-1* promoter region. Extensive methylation is a characteristic of a more advanced CRC while partial methylation is the feature of an earlier stage of CRC. These results indicate that the methylation of *DLC-1* promoter runs through the whole course of colorectal tumorigenesis.

These pieces of evidence indicate that the epigenetic mechanism is a driving factor of *DLC-1* transcriptional silencing and may be involved in the tumorigenesis of CRC as an independent risk factor.

*DLC-1* methylation levels were also found to be significantly associated with Duke's stage. The methylation levels were significantly higher in advanced stage (Stages C and D) tumors, which indicated the role of *DLC-1* in the CRC progression. This result is consistent with previous findings, which showed the methylation status of *DLC-1* was related to TNM stages [23]. Jin et al. [24] reported that the knocking down of *DLC-1* transcriptional expression by RNAi resulted in the promotion of LoVo CRC cell proliferation, migration, and cell cycle progression, that is critical for tumor growth and metastasis. Thus, our results further imply that methylation of *DLC-1* promoter is a potentially biomarker for prognosis evaluation of CRC.

KRAS oncogene is a guanine nucleotide-binding protein with GTPase activity that is involved in signal transduction. In this study, we confirmed that the extensive *DLC-1* methylation was associated with the *KRAS* mutations in CRCs but not in adenomas. Our previous study showed that *DLC-1* methylation significantly correlated with *PIK3CA* mutations in Paget's disease [25]. These findings highlighted the interaction between genetic and epigenetic alterations in CRC, although the mechanism underlying this phenomenon requires further study. On the other hand, overactivation of certain oncogenic pathways is known to affect the activity of methyltransferases and the regulation of gene transcription, thus possibly affecting components of the MAPK pathway, such as RAS, RAF, MEK, and ERK [26, 27]. Data from *KRAS* transformation studies suggest that activated *KRAS* promotes P16 methylation [28]. Stable transformation of colon cancer cells with *KRAS* increased DNA methyltransferase activity and P16 gene methylation [29]. Collectively, the results from our study and previous work by others suggest epigenetic alterations of *DLC-1* might occur as a consequence of overactivation of the oncogenic pathway in cancer.

In conclusion, the current data suggested that methylation-induced epigenetic silencing of *DLC-1* is involved in the colorectal tumorigenesis and has a strong correlation with the Duke's stage, and with *KRAS* mutation. Quantitative detection of *DLC-1* promoter methylation may have a promising clinical significance in the evaluation of CRC patients and in the management of the disease.

## Figures and Tables

**Table 1:** Primers for MS-HRMA, HRMA, and real-time PCR.

Usage	Primer	Sequence (5′–3′)	Amplicon (bp)
MS-HRMA	MS-HRM-F	TCGTTACGGTTTTAGAAAGAAA	134
	MS-HRM-R	TTCGCTCCCAACCAAAACATAA	

HRMA	KRAS-F	CTGAATATAAACTTGTGGTAGTTGGA	59
	KRAS-R	TATCGTCAAGGCACTCTTGC	
	BRAF-F	GGTGATTTTGGTCTAGCTACAG	147
	BRAF-R	AGTAACTCAGCAGCATCTCAGG	

Real-time PCR	GAPDH-F	GAAGGTGAAGGTCGGAGTCA	226
	GAPDH-R	GAAGATGGTGATGGGATTTC	
	DLC-1-F	ACCTGATCACGCAACAGTGAAACA	191
	DLC-1-R	AGACGCCTGCATAGAGCCTCA	

**Table 2:** Patients' basic clinical characteristics and molecular alternation information.

	Normal mucosa	Adenomas	Carcinomas	*P* value
Age (y)				
<60	19	19	33	0.631
≥60	29	38	47	
Gender				
Female	18	28	45	0.121
Male	30	29	35	
*DLC-1* methylation				
Nonmethylation	43	31	32	<0.001
Partial methylation	5	18	19	
Extensive methylation	0	8	29	
*DLC-1* mRNA expression				
Abundant	41	27	17	<0.001
Reduction	7	30	63	
*KRAS*				
Wild-type	44	47	48	<0.001
Mutation	4	10	32	
*BRAF*				
Wild-type	47	55	78	0.894
Mutation	1	2	2	

**Table 3:** Methylation status, pathological features, and molecular alternations in CRCs and adenomas.

	CRCs				Adenomas			
	Non methylation	Partial methylation	Extensive methylation	*P* value	Non methylation	Partial methylation	Extensive methylation	*P* value
Age (y)								
<60	14	4	15	0.098	8	8	3	0.397
≥60	18	15	14		23	10	5	
Gender								
Female	14	11	20	0.139	13	11	4	0.433
Male	18	8	9		18	7	4	
Location								
Distal	17	15	16	0.151	23	15	6	0.829
Proximal	15	4	13		8	3	2	
Dukes' stage								
A + B	23	7	7	0.004				
C	4	5	8					
D	5	7	14					
Differentiation								
Well and moderately	19	9	11	0.259				
Poorly	13	10	18					
Size								
<1 cm	14	7	13	0.88	14	6	2	0.598
≥1 cm	18	12	16		17	12	6	
*KRAS* mutation status								
Wild-type	24	13	11	0.01	26	14	7	0.889
Mutation	8	6	18		5	4	1	

**Table 4:** Correlation between *DLC-1* mRNA expression and promoter methylation status in adenomas and CRCs.

mRNA expression	Unmethylation and Partial methylation (<10%)	Extensive methylation (≥10%)	*P* value
Reduced	62	31	0.022
Abundant	38	6	
